# Early prediction of biomass in hybrid rye based on hyperspectral data surpasses genomic predictability in less-related breeding material

**DOI:** 10.1007/s00122-021-03779-1

**Published:** 2021-02-17

**Authors:** Rodrigo José Galán, Angela-Maria Bernal-Vasquez, Christian Jebsen, Hans-Peter Piepho, Patrick Thorwarth, Philipp Steffan, Andres Gordillo, Thomas Miedaner

**Affiliations:** 1 State Plant Breeding Institute, University of Hohenheim, 70593 Stuttgart, Germany; 2 KWS SAAT SE, Grimsehlstraße 31, 37574 Einbeck, Germany; 3 Biostatistics Unit, Institute of Crop Science, University of Hohenheim, 70593 Stuttgart, Germany; 4 KWS LOCHOW GMBH, Ferdinand-von-Lochow Straße 5, 29303 Bergen, Germany

**Keywords:** Biomass, Genetic relatedness, High-throughput phenotyping, Genomic prediction, Prediction ability, Rye

## Abstract

**Key message:**

Hyperspectral data is a promising complement to genomic data to predict biomass under scenarios of low genetic relatedness. Sufficient environmental connectivity between data used for model training and validation is required.

**Abstract:**

The demand for sustainable sources of biomass is increasing worldwide. The early prediction of biomass via indirect selection of dry matter yield (DMY) based on hyperspectral and/or genomic prediction is crucial to affordably untap the potential of winter rye (*Secale cereale* L.) as a dual-purpose crop. However, this estimation involves multiple genetic backgrounds and genetic relatedness is a crucial factor in genomic selection (GS). To assess the prospect of prediction using reflectance data as a suitable complement to GS for biomass breeding, the influence of trait heritability ($H^{2}$) and genetic relatedness were compared. Models were based on genomic (GBLUP) and hyperspectral reflectance-derived (HBLUP) relationship matrices to predict DMY and other biomass-related traits such as dry matter content (DMC) and fresh matter yield (FMY). For this, 270 elite rye lines from nine interconnected bi-parental families were genotyped using a 10 k-SNP array and phenotyped as testcrosses at four locations in two years (eight environments). From 400 discrete narrow bands (410 nm–993 nm) collected by an uncrewed aerial vehicle (UAV) on two dates in each environment, 32 hyperspectral bands previously selected by Lasso were incorporated into a prediction model. HBLUP showed higher prediction abilities (0.41 – 0.61) than GBLUP (0.14 – 0.28) under a decreased genetic relationship, especially for mid-heritable traits (FMY and DMY), suggesting that HBLUP is much less affected by relatedness and $H^{2}$. However, the predictive power of both models was largely affected by environmental variances. Prediction abilities for DMY were further enhanced (up to 20%) by integrating both matrices and plant height into a bivariate model. Thus, data derived from high-throughput phenotyping emerges as a suitable strategy to efficiently leverage selection gains in biomass rye breeding; however, sufficient environmental connectivity is needed.

**Supplementary Information:**

The online version contains supplementary material available at(10.1007/s00122-021-03779-1) .

## Introduction

Worldwide, the consumption of energy obtained from renewable origins, especially bio-based sources, is rising (World Bioenergy Association 2019). In the European Union (EU), for instance, the share of renewable energy is expected to be between 55 and 75% of the total energy consumption in 2050, increasing in proportion the needs for biomass (European Commission 2011). New policy directives have established sustainability guidelines for bioenergy production (European Union 2010). For example, in Germany, the principal European biogas producer, the permitted share of maize (*Zea mays* L.) as the most common fermentation substrate has been limited to 44% by 2021 (Renewable Energy Sources Act “EEG”, EEG 2017). Thus, suitable alternatives are welcome to diversify maize-based biomass production.

Among the small-grain cereals, winter rye (*Secale cereale* L.) stands out for its vigorous growth and enhanced tolerance to abiotic and biotic stress factors. Europe is the largest rye grower worldwide covering about 81% of the global area with Russia, Poland, and Germany being the main producers (FAO 2019). In a previous study, rye demonstrated its high dry matter yield (DMY) potential even on sandy soils and under drought stress (Galán et al. 2020a). Under these conditions, rye yielded 8.4 t dry matter ha^−1^, and under better environmental conditions, yields were up to 14.7 t dry matter ha^−1^. Rye can, therefore, represent a suitable alternative for biomass production in a variety of agroecological conditions, including areas where the cultivation of other cereal crops would not be competitive (Geiger and Miedaner 2009). Considering that three quarters of the rye harvest is used for non-food purposes, rye appears as a sustainable alternative source of biomass (Geiger and Miedaner 2009; Miedaner et al. 2012).

In Germany, only 4 rye varieties are currently registered for whole plant silage (Bundessortenamt 2019). Rye is, however, mainly bred for grain yield (GY; Haffke et al. 2014) which is, in the breeding scheme proposed here, already assessed in the first year of general combining ability testing (GCA-1), generally sharing only less-related genotypes over the years (Suppl. Fig. 1). Then, within each selection cycle, a selected fraction of GCA-1 is re-evaluated for GY and additionally for DMY by destructive methods in duplicated GCA-2 experiments the following year, mainly due to the high costs of assessing DMY in a large GCA-1 population. At these first selection stages, the enhancement of DMY is, therefore, heavily dependent on the adequate exploitation of indirect selection (Falconer and Mackay 1996).

Higher selection gains have been reported when plant height (PH) was used as a secondary trait instead of GY to indirectly estimate DMY in hybrid rye (Haffke et al. 2014; Galán et al. 2020a). Recently, multi-kernel models jointly using reflectance and genomic data as alternative sources of information and bivariate models including also the routinely assessed PH were suggested as superior strategies to leverage rye as a dual-purpose crop in an affordable manner for the breeder (Galán et al. 2020b). By this, the available genetic variation present in the GCA-1 population may be better exploited without the need to duplicate these large-scale trials and, therefore, the selection gain for DMY could be further enhanced. In consequence, fewer and superior DMY-genotypes being tested in GCA-1 trials could be identified, reducing the amount of capital, time, and labor needed to conduct the destructive sampling of DMY in GCA-2 trials. In this context, the non-destructive assessment of DMY at earlier stages arises as a crucial prerequisite.

Imaging-based phenotyping quantitatively measures the interaction (e.g., absorbance, reflectance, or transmittance of photons) between the incident light and plant tissues, which at specific regions of the electromagnetic spectrum is linked to a wide range of morphological and physio-chemical canopy properties (Li et al. 2014). As observed by Rincent et al. (2018), this interaction is mainly mediated by the chemical composition of the tissues, which is itself determined by endophenotypes, intermediate molecular phenotypes associated with a quantitative trait (Mackay et al. 2009), and genetics. Thus, based on reflectance data, high-throughput phenotyping (HTP) can acquire a considerable amount of detailed phenotypic information of key traits from a large number of genotypes, emerging as a valuable breeding tool (Montes et al. 2007; Cabrera-Bosquet et al. 2012; Würschum 2019).

Examples of the application of HTP in plant breeding are among others, the estimation of above-ground biomass (Babar et al. 2006; Montes et al. 2011; Busemeyer et al. 2013; Fu et al. 2014; Barmeier and Schmidhalter 2017; Yue et al. 2017, 2018) as well as GY, plant responses to biotic and abiotic stress, nitrogen use efficiency, nutrient status, early plant vigor, seeds quality traits, leaf physiology and biochesmistry, vegetation cover fraction, and leaf area index (reviewed by Fahlgren et al. 2015; Yang et al. 2017; Würschum 2019). Therefore, it has been proposed to remotely phenotype large breeding populations in a reliable and cost-effective manner (Furbank and Tester 2011; White et al. 2012). HTP platforms, including uncrewed aerial vehicles (UAVs) such as drones mounted with hyperspectral cameras, can simultaneously collect hundreds of high-resolution images, screening the electromagnetic spectrum (from 400 up to 2500 nm) in a continuous mode (Araus and Cairns 2014). Consequently, this noninvasive technology represents a valuable tool for the improvement of complex traits (Finkel 2009; Fiorani and Schurr 2013).

Genome-wide molecular markers integrated into genomic selection (Meuwissen et al. 2001) have been successfully applied in several study cases in hybrid rye breeding for relevant traits, e.g., GY and GY components (Auinger et al. 2016; Bernal-Vasquez et al. 2017; Miedaner et al. 2019). Moreover, in previous studies, reflectance fingerprints recorded by HTP platforms represented a valuable tool to improve the prediction ability of DMY in hybrid rye of models based on agronomic (Galán et al. 2020a) and genomic information (Galán et al. 2020b). These studies have shown the benefits of integrating hyperspectral and molecular information for predicting DMY of unphenotyped candidates within single or closely related populations. The proposed models were cross-validated, where rye lines derived from the same cross were randomly allocated to the training (TRN) or validation (VAL) sets. Considering the breeding scheme at hand, where DMY is tested at later stages, predictions of candidates of subsequent selection cycles, where TRN and VAL correspond to different, largely independent genetic backgrounds, would be of utmost interest. This “across-cycles” prediction would allow, for instance, estimating the DMY performance of GCA-1 candidates (being tested only for GY at this stage) by training the model with GCA-2 phenotypic data from one or several previous selection cycles (Suppl. Fig. 1). It is under these scenarios where the largest contribution of predictive breeding towards an affordable dual-purpose rye breeding program is expected. If the data available consist of multiple connected cycles, breeders could consider to combine them to improve the predictive power of models (Auinger et al. 2016).

However, the predictive power of GS critically depends on a close relationship between TRN and VAL (Habier et al. 2007; Miedaner et al. 2019). Reduced or even negative prediction accuracies were reported for GS among less related bi- and multiparental families in several crops, including wheat (Herter et al. 2019), maize (Riedelsheimer et al. 2013; Lehermeier et al. 2014), sugar beet (Würschum et al. 2013), and barley (Thorwarth et al. 2017). Similarly, genomic prediction models showed modest prediction ability for complex traits in rye (e.g., GY) when applied between bi-parental families even though they were connected by a common parental line (Wang et al. 2014). Here, the question of whether alternative or complementary approaches to GS for leveraging prediction accuracies across less connected datasets emerges as highly relevant for biomass breeding in rye.

The aim of our study was, therefore, to answer this question by evaluating and comparing genomic- and hyperspectral-enabled predictions for three biomass-related traits (DMY, FMY, and DMC) in rye under a varying degree of relatedness between TRN and VAL. Additionally, the advantages of combining different sources of information in multi-kernel and bivariate models to leverage the prediction of DMY were evaluated. We employed 270 winter rye lines from nine interconnected bi-parental families, including their parental components tested as testcrosses in 8 environments (= location-year combination). While keeping the TRN size constant, our specific objectives were to perform (1) prediction of progenies from half-sib and unrelated parents, (2) prediction using only progenies from unrelated parents, and (3) prediction of new progenies in a new environment.

## Materials and methods

### Plant materials, field experiments, hyperspectral and molecular data

The plant materials, field experiments, molecular and hyperspectral data analyzed in the present study have been described before in detail by Galán et al. (2020b). In short, ten diverse parental lines of the Petkus (seed parent) gene pool were crossed following a single-round robin design (Verhoeven et al. 2006). F1 progenies were derived from each of the chain crosses, i.e., line 1 × line 2, line 2 × line 3, …, line 10 × line 1. After self-fertilization of single F1 plants for four consecutive years (S_4_ generation), 264 recombinant inbred lines (RILs) were obtained. The ten bi-parental families ranged from 4 up to 32 RILs (Supp. Fig. S2) and were clearly distinct in a principal component analysis (PCA) based on molecular data with little overlap between unrelated crosses in the first two dimensions (Supp. Fig. S3). A total of 274 three-way hybrids [(A • B) × C] were produced from the cross of these 264 RILs and their ten parental components with a single-cross tester from the opposite (pollinator) gene pool. They were evaluated in two adjacent trials laid out as a resolvable incomplete block design (*α*-lattice design) with two replicates in 2017 and 2018 at each of four ecologically different locations (Bernburg, Petkus, Wohlde and Prislich) in Northern Germany (i.e., eight location-year combinations hereafter referred as “environments”). All 274 testcrosses were used for estimating means, variance components, and heritabilities (Table 2), whereas 4 genotypes were not considered for prediction modeling as described in later sections. Plots were harvested at the late milk stage (Meier 1997) to get the respective fresh biomass yield (FMY, dt ha^−1^) per plot. During harvest, representative samples of about 1000 g were weighed from each plot and oven-dried to a constant weight at 110 °C. Dry matter content (DMC, %) was determined by weight differences. Then, DMY (dt ha^−1^) per plot was estimated as DMY = FMY × DMC/100. Also, PH (cm) was recorded at each plot.

During the grain-filling stage, an UAV (Camflight FX8HL, Sandnes, Norway) fitted with a hyperspectral camera (HySpex Mjolnir V-1240, Skedsmokorset, Norway) collected reflectance fingerprints consisting of 400 bands (410 nm – 993 nm) for all genotypes in all environments. The UAV flew at about 25 m above plots, around solar noontime two times per environment (except in Bernburg 2017 where only one flight took place). Then each plot was identified on the obtained images by a polygon. Raw data were radiometrically calibrated (HySpex PostProcessor Version 1.2) and normalized based on the incident sunlight as well as orthorectified and georeferenced via the PARGE Software (ReSe Applications LLC, Wil, Switzerland). Lastly, all data points within each wavelength and polygon were averaged, resulting in one spectrum per plot. Then, these data were transferred to a tabular data frame, including the computed reflectance values of all bands for all genotypes for further analysis.

The 264 RILs and their ten parental components were also genotyped with an Illumina INFINIUM chip with 9,963 single nucleotide polymorphisms (SNPs) assays (KWS SAAT SE & Co. KG, Einbeck, Germany). Data quality analysis consisted of the exclusion of SNPs showing more than 10% missing values or a minor allele frequency (MAF) < 0.05. Missing values in the remaining data were then imputed by the software Linkimpute (Money et al. 2015). Then, data were again screened for MAF < 0.05. After this procedure, 6,420 markers remained for further analyses.

### Phenotypic data analysis

The analyses were based on adjusted entry means (best linear unbiased estimators, BLUEs) for all agronomic traits estimated within and across environments for subsequent incorporation into prediction models. The combined analysis across environments as well as the data adjustment within single environments were conducted following model (1) and model (2) from Galán et al. (2020b), respectively. The full model can also be found in the Supplementary File 1. For the analysis across environments within the same year, the year main effect and corresponding interactions with genotypes were dropped from the mixed model. Phenotypic data were filtered for outliers at the trial level using the Bonferroni-Holm test (Bernal-Vasquez et al. 2016). Bands were deleted from plots identified as an outlier for DMY.

### Stage-wise procedure for biomass traits prediction

The incorporation of genomic and hyperspectral data for predicting DMY, FMY, and DMC was conducted by a three-stage procedure (Piepho et al. 2012). This analysis, together with the corresponding linear mixed and prediction models employed at each stage, was previously described in detail in Galán et al. (2020b). All statistical analyses were performed within the R-environment v. 3.4.4 (R Core Team 2018).

In the first stage of this analysis, bands were adjusted across flight dates per environment. Then, the obtained adjusted entry means (BLUEs) were used in the second stage for the estimation of BLUEs per genotypes across environments. At this second stage, heritability (${H}^{2}$) was estimated for all agronomic traits and each band across environments as

$$ H^{2} = { }\frac{{{\upsigma }_{{\text{g }}}^{2} }}{{{\upsigma }_{{\text{g}}}^{2} + \frac{{\overline{v}}}{2}}} $$

where $\overline{v}$ is the mean variance of a difference of two adjusted genotype means (BLUEs) estimated for phenotypic and hyperspectral data (Piepho and Möhring 2007). BLUEs of genotypes were calculated with the software package *ASReml-R* v. 3.0 (Gilmour et al. 2009).

In the third stage, the phenotypic and hyperspectral BLUEs were used for fitting prediction models to estimate best linear unbiased predictions (BLUP) of genotypic effects for each agronomic trait based on genetic and hyperspectral data. Two single-kernel prediction models were fitted with genetic (genomic BLUP, GBLUP) or hyperspectral (hyperspectral BLUP, HBLUP) data with *n* = 270 individuals, based on the *m* = 6,420 conserved SNP markers or *b* = 32 bands, respectively.

For GBLUP, the random genetic values (effects) were estimated based on genetic data incorporated into **G**, a genomic additive relationship matrix (Habier et al. 2013). **G** was calculated with the *synbreed* package (Wimmer et al. 2012) in R according to the “method I” of VanRaden (VanRaden 2008) as $G = \frac{{ZZ^{\prime }  }}{{2\sum p_{i} (1 - p_{i})}}$, where ${\text{Z = M - P}}$, ${\text{M}}$ is the *n* × *m* marker matrix reflecting the SNP genotype of *n*th individual at the *m*th SNP position the of alleles coded as 0, 1, and 2 for A_1_A_1_, A_1_A_2_, and A_2_A_2_, respectively, ${\text{P}}$ contains a *n* × *m* matrix of allele frequencies multiplied by 2, $p_{i }$ is the allele frequency of the *i*th allele. For the prediction scenario S2 (described below), the GBLUP model was adapted from the model (7) in Bernal-Vasquez et al. (2017) as

1 $$ y = Xb + Z_{g} u_{g} + Z_{ge} u_{ge} + e $$

where ${\varvec{y}}$ is the vector of BLUEs of genotype trait values obtained from within-environments, ${\varvec{X}}$ is the design matrix of the environments,${ }{\varvec{\beta}}$ is the vector of environments effects, ${\varvec{Z}}_{{\varvec{g}}}$ is the marker matrix for genotypes, and ${\varvec{u}}_{{\varvec{g}}}$ the vector of marker effects. The genotype-by-environment effects is modelled by ${\varvec{w}} = {\varvec{Z}}_{{{\varvec{ge}}}} {\varvec{u}}_{{{\varvec{ge}}}}$, with ${\varvec{Z}}_{{{\varvec{ge}}}}$ standing for the marker matrix for genotypes-by-environment effects and ${\varvec{u}}_{{{\varvec{ge}}}}$ the vector of marker-by-environment effects with variance $var\left( {{\varvec{u}}_{{{\varvec{ge}}}} } \right) = {\mathbf{I}}\sigma_{ge}^{2}$, thus $var\left( {\varvec{w}} \right) = {\varvec{Z}}_{{{\varvec{ge}}}} {\varvec{Z}}_{{{\varvec{ge}}}}^{{\varvec{T}}} \sigma_{ge}^{2}$. ${\varvec{Z}}_{{{\varvec{ge}}}}$ is a block-diagonal matrix with blocks given by the marker coefficient matrices of genotypes in a given environment $\left( {Z_{{ge_{r} }} } \right)$ and for the eight environments considered in the present study, it can be defined as $$ \left( {\begin{array}{*{20}c}    {Z_{{ge_{1} }} } & 0 & 0  \\    0 &  \ddots  & 0  \\    0 & 0 & {Z_{{ge_{8} }} }  \\   \end{array} } \right) $$. The variance of ${\varvec{w}}$ stands for the linear structure of the genotype-by-environment variance–covariance matrix with the covariance of two genotypes within the same environment depending on the similarity in their marker profiles (Piepho 2009). Since the covariance among different environments is zero, any covariance between environments is captured by ${\varvec{Z}}_{{\varvec{g}}}$.

As a measure of the genetic similarity among all *n* candidates, the Pearson’s coefficients of correlation among rows of ${\text{M}}$ were calculated. Based on their SNP alleles, this genomic correlation ($r_{GC}$) reflects the correlation pattern among individuals (Riedelsheimer et al. 2013). In contrast, for HBLUP the estimation of the random genetic values was based on reflectance data integrated into the hyperspectral reflectance-based relationship matrix **H** defined as ${\varvec{H = DD}}^{\prime }$, where ${\varvec{D}}$ is a *n* × *b* hyperspectral matrix of the standardized BLUEs of the bands, with *b* = 32. These 32 bands belong to the visible spectrum (VS) and the infrared radiation (IR), and they were selected in a previous study (Galán et al. 2020b) using the least absolute shrinkage and selection operator (Lasso; Tibshirani 1996) for reducing the multicollinearity observed among continuos bands and increasing, therefore, the predictive power of reflectance-based models. Following the same procedure as described before for $r_{GC}$, a second correlation ($r_{HC}$) among tested genotypes was developed based on hyperspectral data incorporated into **H´,** which was derived from *b* = 400 available bands. By this, the correlation pattern among lines was estimated based on their unique reflectance fingerprints along the whole spectrum.

For the prediction scenario S1B (described below), the advantages of integrating different information sources to improve the predictive ability of DMY were assessed following the procedures described in Galán et al. (2020b). For this, genetic and hyperspectral data were combined in a multi-kernel prediction model (G + H), which was further extended to a bivariate model (Bivariate_G + H) by incorporating PH a as predictor.

All third-stage prediction models were fitted using the *sommer* package in R (Covarrubias-Pazaran 2016), except model (1), which was fitted within the R package *ASReml-R* v. 3.0 (Gilmour et al. 2009).

### Prediction schemes

To address the objectives of the present study, nine bi-parental families with a size of 24 to 32 individuals (Suppl. Fig. S2) and their parental components were divided into TRN and VAL following different schemes. The family 4 × 5 was not considered due to its reduced size (*n* = 4). The TRN composition varied in a controlled manner for testing the effect of the relatedness between this set and VAL on a genotypic level (S1) and both genotypic and environmental levels simultaneously (S2). An overview of the different prediction schemes is given in Table 1.

**Table 1:** Overview over the validation scenarios (TRN, training set; VAL, validation set; UR, Unrelated; HS, Half sibs; FS, Full sibs; P: Parental lines)

Name	TRN^a^	VAL	Relationship	No. environments sampled^b^	
				TRN	VAL
S1CV	8 random folds	1 Random fold	UR + HS + FS + P	8	8
S1A	8 families	1 Family	UR + HS	8	8
S1B	6 or 7 families	1 Family	UR	8	8
S2	6 or 7 families	1 Family	UR	7	1

^a^The TRN size remained constant across all S1-scenarios (*n* = 174)

^b^Corresponds to combined years predictions

In S1, three different scenarios were analyzed, namely S1CV, S1A, and S1B, which have a decreasing genotypic relationship between TRN and VAL. Scenario S1CV consisted in ninefold cross-validation (CV) of the whole data set (the nine bi-parental families and their parental components), with eight folds were used for model training and the remaining fold for validation purposes. In contrast, in S1A and S1B, a leave-one-out (LOO) family validation scheme was followed. Here, TRN ranged from six to eight bi-parental families, and VAL consisted of single families with variable size. Whereas in S1A half-sibs (HS) and unrelated lines (UR) were sampled in TRN, in S1B, it included only UR. Parental lines of VAL were available for model training only in S1CV. In contrast, under S1A and S1B, the parents of the validation family were excluded from TRN. The remaining eight parents were considered as UR and could be incorporated accordingly. Genotypes were classified as “unrelated” to distinguish this cross type from FS and HS and, therefore, this term does not have the same meaning as in a population genetics.

To avoid the influence of the TRN size on the prediction ability, for all three scenarios, the TRN size was fixed to 174, which was the largest possible common size among scenarios. If TRN was initially larger than 174, a random sampling without replacement was conducted among possible candidates in order to achieve the targeted size of 174. This procedure was repeated 9,000 times, each repetition consisting of a random composition of TRN to assess model error. The phenotypic and hyperspectral data included in S1 validation scenarios were adjusted across the same four (within-year analysis) or eight (combined years analysis) environments.

In S2, the predictive power of models was assessed by a LOO family validation scheme, as described above for S1. An important difference between S1 and S2 scenarios, is that for S2, data not connected to TRN, either by environments nor by genotypes, was used as VAL. For this, data for model training was collected on UR from six to seven bi-parental families at three or seven environments, while validation data came from single families of variable size evaluated at a fourth or eighth disconnected environment, for the within-year or combined years predictions, respectively.

For all prediction schemes, prediction ability was assessed as the Pearson’s coefficients of correlation $r$ between predicted breeding values and observed BLUEs derived from the combined analysis across environments for S1 and data adjustment within single environments for S2.

## Results

### Population structure, phenotypic and hyperspectral data analysis

**Fig. 1:**
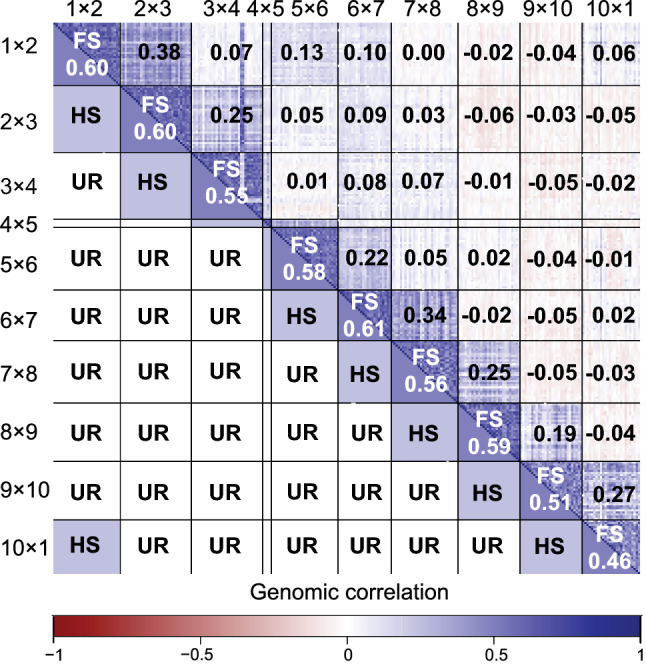
Heatmap showing the relatedness based on prior pedigree information (below diagonal) and the genomic correlation (above diagonal) among 264 rye lines distributed among ten bi-parental families. The numbers in the blocks refer to average genomic correlations between all pairs of individuals. FS, full sibs; HS, half sibs; UR, unrelated (color figure online)

**Fig. 2:**
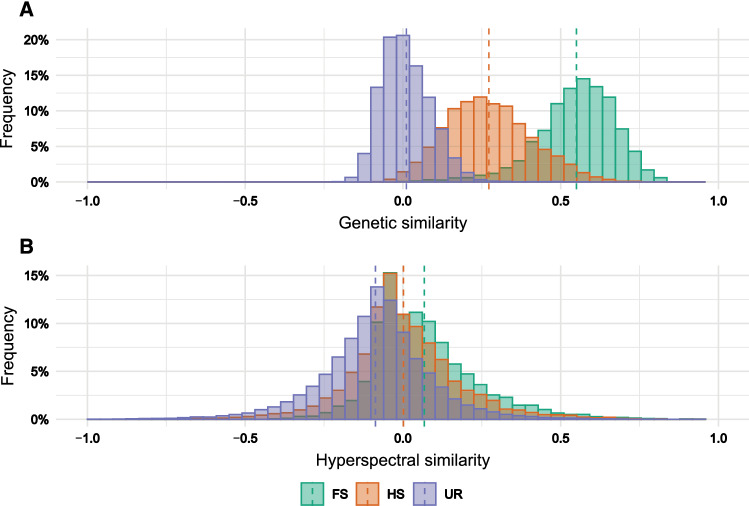
Histograms of (A) genetic similarity and (B) hyperspectral similarity for full sibs (FS), half sibs (HS), and unrelated (color figure online)

The population showed a genomic correlation pattern (Fig. 1), which clearly reproduces the SRR mating design used in the present study (Suppl. Fig. S2). Thus, the mean genomic relationship among full sibs (FS), half sibs (HS), and unrelated lines (UR) followed the expected decay based on prior pedigree information (Fig. 2a). Nevertheless, a substantial overlap between the $r_{GC}$ values from HS with FS and UR was observed. The mean $r_{GC}$ among the nine FS families was 0.55, with a range from 0.61 to 0.46. For HS, the average $r_{GC}$ was 0.27, almost the mean between FS and UR (0.01). The highest $r_{GC}$ among HS was 0.38, while the smallest correlation coefficient was 0.06. Among UR, $r_{GC}$ ranged from 0.04 to − 0.04. Interestingly, no clear distinction among lines could be drawn based on reflectance data (Fig. 2b). The $r_{HC}$ for FS, HS, and UR was close to zero, with mean estimates equal to 0.07, zero, and − 0.09, respectively.

In 2017, FMY and DMY had higher mean estimates than in 2018 (Table 2). In the first year, these values were 355.96 dt ha^−1^ and 124.18 dt ha^−1^, respectively, whereas in the second year, they dropped correspondingly to 304.82 dt ha^−1^ and 114.68 dt ha^−1^. The contrary was observed for DMC, which showed a higher mean in 2018 (38.84%) than in 2017 (35.24%). The estimated genotypic variance ($\sigma_{g }^{2}$) was significantly greater than zero (*p* < 0.001) for all traits. With one minor exception, the same holds for the genotype-by-location interaction ($\sigma_{gl }^{2}$) and genotype-by-location-by-year interaction ($\sigma_{gly }^{2}$) variances. The estimates of $H^{2}$ were in general higher in 2017 than in 2018. DMC displayed the higher $H^{2}$ estimates, which ranged from 0.70 to 0.81, whereas $H^{2}$ for FMY and DMY varied from 0.46 to 0.56. Across the analyzed hyperspectral spectrum, $H^{2}$ was highly heterogeneous. The mean value across the 32 selected hyperspectral bands (Suppl. F4) was higher when both years were analyzed together ($H^{2} = 0.63$), followed by 2018 ($H^{2} = 0.54$) and 2017 ($H^{2} = 0.43$). Mean correlations with agronomic traits (considering absolute values) were rather low for all traits ($r \le$ |0.16|) with relatively broad ranges (up to $r \le$ |0.41|, Suppl. Fig. S4). FMY mostly displayed the highest correlation estimates, followed by DMY and DMC.

**Table 2:** Means, ranges, estimates of variance components (genotypic, $\sigma_{{\text{g }}}^{2}$; genotype-by-location interaction, $\sigma_{{gl{ }}}^{2}$; genotype-by-year-by-location interaction, $\sigma_{gyl}^{2}$; and residual error $\sigma_{{\varepsilon { }}}^{2}$), heritabilities $H^{2}$ determined from 274 winter rye hybrids assessed in two years, which were individually or combined analyzed

Trait^a^	Means and ranges			Variance components				$H^{2}$
	Mean	Min	Max	${\sigma }_{g }^{2}$	${\sigma }_{gl }^{2}$	${\sigma }_{gly }^{2}$	${\sigma }_{\varepsilon }^{2}$	
2017								
FMY (dt ha^−−1^)	355.96	332.85	386.17	41.61***	43.76***	–	190.02	0.56
DMY (dt ha^−1^)	124.18	116.63	131.74	4.97***	6.04***	–	16.62	0.53
DMC (%)	35.24	34.02	37.06	0.23***	0,04***	–	0.36	0.80
2018								
FMY (dt ha^−1^)	304.82	284.92	323.87	25.80***	27.15***	–	213.14	0.46
DMY (dt ha^−1^)	114.68	105.85	122.31	5.85***	6.79***	–	26.22	0.54
DMC (%)	38.84	37.21	40.62	0.27***	0.13***	–	1.51	0.70
Combined								
FMY (dt ha^−1^)	330.68	312.29	351.91	21.31***	15.15***	19.04***	203.13	0.47
DMY (dt ha^−1^)	119.48	113.31	126.33	3.41***	2.54***	3.64***	21.49	0.50
DMC (%)	37.02	35.74	38.45	0.23***	0.02	0.07***	0.94	0.81

^a^Traits are fresh matter yield (FMY), dry matter yield (DMY), and dry matter content (DMC)

^***^Significant at the 0.001 probability level

### Prediction abilities under declining genotypic relationships (S1)

**Fig. 3:**
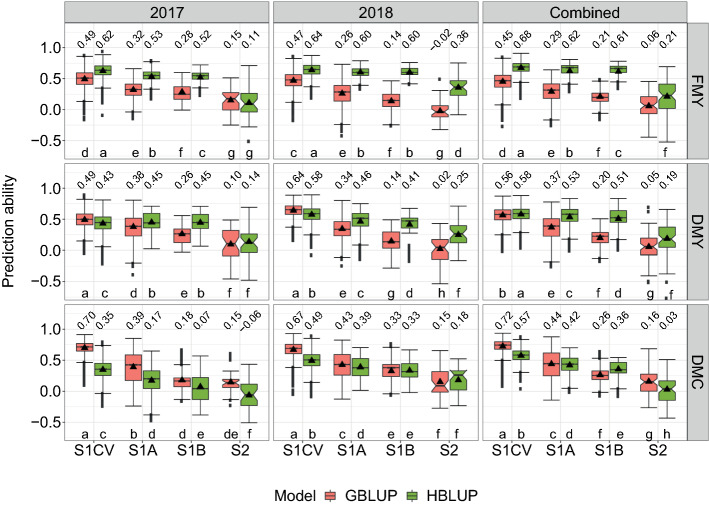
Prediction abilities for fresh matter yield (FMY), dry matter yield (DMY), and dry matter content (DMC) of genomic (GBLUP) and hyperspectral (HBLUP) best linear unbiased predictions under four different validation schemes assessed across two years (2017 and 2018), which were individually and combined analyzed. Mean values are shown above each box plot and by black triangles and are significantly different, within each subplot, when no letter in common is shared (Tukey's honestly significant difference test; α = 0.01) (color figure online)

**Fig. 4:**
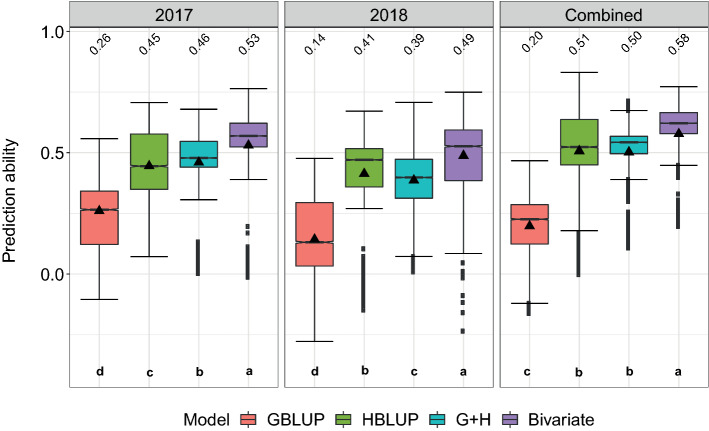
Prediction abilities for dry matter yield of single-kernel (Genomic best linear unbiased predictor, GBLUP and Hyperspectral best linear unbiased predictor, HBLUP), multi-kernel (G+H), and bivariate (Bivariate_G+H) models assessed across two years (2017 and 2018), which were individually and combined analyzed. Models were tested under validation scenario S1B. Mean values are shown above each box plot and by black triangles and are significantly different, within each subplot, when no letter in common is shared (Tukey's honestly significant difference test; α = 0.01) (color figure online)

Overall, HBLUP was significantly more accurate than GBLUP for FMY and DMY, while the opposite was observed for DMC (Fig. 3). Combining the data across years was beneficial for HBLUP for all traits, and in the case of GBLUP only for DMC, while for FMY and DMY, GBLUP was mostly more accurate within single-year analysis. The highest prediction abilities for all models and traits were observed under validation scenario S1CV, which has the closest relationship between genotypes used for model training and validation, followed by scenarios S1A and S1B (Fig. 3). However, the impact of a reduced degree of genetic relatedness between TRN and VAL on the prediction ability was unequal between models. Interestingly, the reduction of the predictive power of GBLUP was considerably higher than for HBLUP when validated on less related sets. For instance, under S1CV for DMY adjusted across years (Fig. 3), GBLUP showed a prediction ability of 0.56, while it dropped to 0.37 and 0.20 under scenarios S1A and S1B, respectively. This represents a decay of about one third and two thirds, respectively. In contrast, the prediction abilities of HBLUP were more stable since they ranged between 0.58 (S1CV) and 0.51 (S1B). Thus, HBLUP retained about 90% of the predicted power shown under S1CV when predicting UR genotypes (S1B). A similar trend was observed for all other traits within as well as across years. The prediction of DMY in validation scenario S1B could be further enhanced by a bivariate model combining hyperspectral and genomic data as well as PH (up to 0.58, Fig. 4). In contrast, the predictive power of the multi-kernel model was very similar to the achieved by HBLUP, although a slight reduction in the variability of the predictions was observed in 2017 and combined-years analysis.

### Predicting environmentally and genetically unconnected candidates (S2)

In the present study, the prediction models were additionally trained on unrelated data, either at a genotypic or environmental level, with the data used for model validation (scenario S2). Under S2, the prediction abilities of all models for all traits was significantly lower and displayed broader ranges when compared to their performance under S1 (Fig. 3). Under S2, HBLUP was also mostly more accurate than GBLUP for FMY and DMY, while the opposite was observed for DMC. Ranges of mean prediction ability of HBLUP for FMY, DMY, and DMC were from 0.11 to 0.36, from 0.14 to 0.25, and from -0.06 to 0.18, respectively, while for GBLUP ranges lay between − 0.02 to 0.15, 0.02 to 0.10, and 0.15 to 0.16, respectively. In contrast to S1, no clear benefits of combining the data collected across years were observed under the S2 validation scheme.

## Discussion

The accurate prediction of biomass at early stages via indirect selection for DMY based on GY trials is a fundamental requirement for the implementation of a resource-efficient dual-purpose breeding program in rye. In this way, the entire genetic variance could be exploited, leveraging the expected selection gain. In our breeding program, each year represents a new selection cycle, where genotypes with different genetic backgrounds are evaluated in new GCA trials. The prediction of subsequent selection cycles implies an additional challenge since the data used for model training and validation are highly unconnected. Nevertheless, it is mainly under this scenario that breeding programs can benefit the most because the biomass improvement can be conducted at the first stage of testcross evaluation without an increase of the number of field plots. The objective of this study was, therefore, to assess and compare the prediction ability of genetic and hyperspectral data under varying genetic relationships between the training and validation sets.

## Influence of the genetic composition of the TRN and traits characteristics

The degree of relatedness between individuals used for model training (TRN) and validation (VAL) directly influenced the prediction ability of all models; however, this impact was remarkably lower for HBLUP than for GBLUP (Fig. 3). The prediction abilities observed under scenario S1CV can be considered as an upper limit, where model training is performed across FS, HS, UR, and parental lines of genotypes used for model validation. Then, a systematic reduction in the predictive performance of all models accompanied the exclusion of genotypes genetically closest to VAL. The exclusion of FS and parental lines from TRN (S1A) represented, averaged across single and combined years analyzes and traits, a reduction of about 40% on the performance of GBLUP, while the further removal of HS signified an additional penalization of around 20%. The larger drop in the prediction abilities observed for S1A compared to those of S1B can be explained by the asymmetrical relevance of using closest relatives for genomic model training (Albrecht et al. 2011; Technow et al. 2014; Juliana et al. 2019). In contrast to GBLUP, the penalization observed for HBLUP in S1A was, on average, only nearly 15% and an additional 6% in S1B, allowing this model to show the highest prediction abilities between the single-kernel models in these scenarios. Model performance was also dissimilar across traits. GBLUP showed mostly the higher abilities for DMC, whereas HBLUP performed better for FMY and DMY. The differences in predictive abilities are most likely a consequence of both trait $H^{2}$ and the different information sources used by GS and reflectance-based models.

To adequately predict the performance of untested candidates, genomic models exploit the genetic relationships between them and individuals whose genotypic and phenotypic information is available, as previously shown in many empirical and simulation-based studies in animal and plant breeding (Habier et al. 2007,2010; Roos et al. 2009; Pszczola et al. 2012; Riedelsheimer et al. 2013; Würschum et al. 2013; Crossa et al. 2014; Lehermeier et al. 2014; Technow et al. 2014; Wang et al. 2014; Thorwarth et al. 2017; Herter et al. 2019). In line with these observations, our results also showed that the predictive power of GS dropped substantially when predictions are made among lowly related populations. For predictions across subsequent cycles in rye, GS could represent a suitable strategy when TRN is represented by aggregated multi-year data from several cycles (Auinger et al. 2016; Bernal-Vasquez et al. 2017). Nevertheless, the authors of these papers concluded that GS still relies heavily on a sufficient relationship between predicted candidates and those used for model training. Selection cycles need, for instance, to be connected by a sufficient number of common ancestors. This prerequisite may not be easily fulfilled in practical rye breeding since subsequent breeding cycles usually are largely unconnected. In addition, the success of GS depends, among others, on trait related factors, such as heritability (Jia and Jannink 2012; Marulanda et al. 2015). Thus, the better and less variable GBLUP performance observed for DMC (Fig. 3) is likely explained by the larger $H^{2}$ estimated for this trait in comparison to FMY and DMY (Table 2).

The reflectance fingerprints of the genotypes were more similar than their allelic status across relationship groups (Fig. 2), suggesting that the information imprinted among the spectrum is less sensitive to genetic distinctiveness among individuals than molecular data. These observations can explain why reflectance data allowed higher prediction abilities than marker data under decreased genetic relationships between TRN and VAL. In contrast to GBLUP, more highly heritable traits were not better predicted by HBLUP. In turn, for HBLUP to perform well, plant canopies should display specific absorption patterns related to some extent to the trait of interest as shown, for instance, by the correlations between the analyzed traits and bands. The most effectively predicted traits (FMY and DMY) showed higher correlations than the lowest predicted trait (DMC, Suppl. Fig. S4). Thus, the higher performance of HBLUP for FMY and DMY might be explained by the higher informativeness of the collected reflectance data for those traits than for DMC. Since the absorption of water and DMC is almost constant across the visible spectrum and the absorbance of these two features starts around 950 nm (Jacquemoud et al. 2000), where our spectrum was from 410 nm to 993 nm, further research could investigate the prospects of HBLUP based on reflectance data beyond 1000 nm to better predict DMC.

Several strategies have been investigated for taking advantage of reflectance data in predictive breeding. Summarizing the reflectance characteristics of plants into simple vegetation indices (VIs) has been proposed to assess vegetation characteristics of interest like grain and biomass yields under different environmental conditions (Xue and Su 2017). However, prediction models benefited the most by the exploitation of whole-spectrum data (Aguate et al. 2017; Montesinos-López et al. 2017b; Krause et al. 2019; Galán et al. 2020b). Recently, highly heritable VIs genetically correlated with the trait of interest such as the Normalized Difference Vegetation Index (NDVI; Rouse et al. 1974; Tucker 1979) and the green NDVI (GNDVI; Gitelson et al. 1996), have been incorporated as secondary traits into multivariate pedigree and genomic prediction models to increase accuracy within the same wheat population and selection cycle (Rutkoski et al. 2016; Sun et al. 2017) as well as across selection cycles composed by closely related populations (Sun et al. 2019). Juliana et al. (2019) found that similar multivariate equations were superior to univariate genomic prediction models when predicting across populations and years. Still, the relationship between TRN and VAL was found crucial also for multivariate models, although the populations used for model training and validation were genetically related to some extent, and predictions were made within the same stressed environments. The results of the present study also showed that combining hyperspectral and genomic data in a multi-kernel model yielded only limited advantages over HBLUP for DMY prediction of less related progenies (Fig. 4). In this context, the prediction ability for DMY could be further increased up to 20% by a bivariate model including also PH. Nevertheless, the performance of G + H and the bivariate model in the present study were lower than when used for DMY prediction of highly related rye progenies, as reported in a previous research (Galán et al. 2020b). These findings reveal, on the one hand, the advantages of incorporating HTP data into prediction routines, and, on the other hand, the limits of GS in the context of across cycle predictions.

## Prediction of new genotypes in untested environments

In validation scenario S1B, UR genotypes were assessed across the same environments (Table 1). In contrast, in S2, unrelated individuals were tested under new environmental conditions, allowing the simultaneous assessment of the genotypic and environmental sampling on the predictive power of marker- and hyperspectral-based models. Predictive abilities in S2 were significantly lower than in S1B, suggesting that predicting the performance of genetically and environmentally highly unconnected individuals is challenging. This is consistent with studies showing that the prediction of new candidates is less accurate when model training is performed without borrowing information of environments correlated to the one used for validation (Crossa et al. 2014; Krause et al. 2019). These poor predictions obtained in S2 might be explained by the substantial genotype-by-environment interactions (G × E) estimated for the predicted traits (Table 2) as well as by the high variability observed for hyperspectral bands among environments, resulting mainly from the extremely different conditions observed between growing seasons as reported in a previous study (Galán et al. 2020b). It seems, therefore, plausible that heterogeneous marker-to-trait and band-to-trait (Montesinos-López et al. 2017a) signals among environments adversely affected the prediction abilities from GBLUP and HBLUP. Therefore, to adequately predict untested genotypes under new environmental conditions, prediction equations need to be extended by environmental and genetic covariates for proper G × E modeling (Piepho 2009; Burgueño et al. 2012; Resende et al. 2020).

A forward-validation approach aims to predict the performance of new genotypes by exploiting the data from previous years (Bernal-Vasquez et al. 2017). Considering our breeding scheme (Suppl. Fig.1), data for model training could be obtained from split GCA-2 trials with biomass and grain yield plots, whereas model validation could be performed on GCA-1 data from a subsequent selection cycle. It should be kept in mind that we need large-drilled plots for biomass model training because this trait cannot be reliably measured on smaller observation plots. As different selection cycles involve new individuals from multiple genetic backgrounds, and usually hardly any common progeny is shared across cycles, the genetic relationship between the data used for model training and validation across cycles is expected to be substantially lower than in within-cycle predictions.

However, data used in across-cycles predictions is environmentally connected because GCA-2 genotypes are tested more intensively in a larger number of locations, within which the same environments as in GCA-1 are typically found. In practical plant breeding, large testing locations within the targeted environment are common, since they are more efficient in terms of logistics, trained personnel requirements, as well as field evaluation and management. In these testing sites, yield trials from different stages are planted next to each other, being reliable, large- scaled training data readily available for model calibration. Thus, scenario S1B mimics this practical situation much better than S2. Our results showed that, in this context, models incorporating hyperspectral data emerge as a promising strategy to achieve superior improvements in DMY in hybrid rye. Still, the relevance of S2 outcomes lies in a consistently unbiased estimation of the prediction abilities of the models (Utz et al. 2000), revealing the high impact of G × E not only on GBLUP but also on HBLUP.

## Conclusions for biomass breeding in hybrid rye

Traditionally, biomass is estimated destructively at an earlier growth stage, preventing grain yield from being evaluated in those same plots. The effective indirect assessment of biomass at the early stages of the breeding program is crucial to entirely untap the potential of rye as a dual-purpose crop affordably. In this sense, prediction models accurately estimating the biomass yield of genotypes of diverse genetic backgrounds across selection cycles represent a valuable tool. In the present study, GBLUP achieved acceptable prediction abilities only for highly heritable traits across closely related individuals. In contrast, HBLUP was substantially less affected by genetic relatedness and trait heritability emerging as a suitable approach for predicting complex traits across highly distinct populations.

Considering that in modern plant breeding genomic information is usually already available before the candidate lines are evaluated as testcrosses in the expensive GCA trials, breeders usually perceive marker and HTP data as a complement, rather than an alternative. Here, HTP offers the possibility of screening large-scale field trials with reduced capital and time expenditures, than conventional methods (e.g. destructive sampling and visual scores). Moreover, combining hyperspectral, genomic, and PH in bivariate models allows more effective DMY predictions of genotypes showing low genetic connectivity to ones used for model training. The bivariate model here presented is flexible and allows the incorporation of GY and other correlated traits to DMY aimig superior predictive power. Nonetheless, by including several predictors, the complexity of the models increases in proportion.

Our results also show that not only GBLUP but also HBLUP was largely affected by G × E interactions, resulting in poor to negligible predictive power when the environments used for model training and validation were different. To fully exploit the advantages of hyperspectral-based models, it is, therefore, highly recommended to incorporate reflectance fingerprints of genotypes collected in the respective environment. Our study demonstrates the capability of hyperspectral-enabled predictions to leverage selection gains to meet the increasing demand for sustainable biomass sources worldwide. Lastly, the prospects of HTP as an economical alternative to traditional biomass sampling are expected to increase in proportion to future improvements in terms of image data acquisition and management.

## Supplementary Information

Supplementary file 1 (PDF 94 kb)

Supplementary file 2 (PDF 499 kb)
