# Supplementary material for: Early prediction of biomass in hybrid rye based on hyperspectral data surpasses genomic predictability in less-related breeding material
Source: Theor Appl Genet. 2021 Feb 17;134(5):1409–22. doi: 10.1007/s00122-021-03779-1 (PMC8081675; doi:10.1007/s00122-021-03779-1)
Supplement: Supplementary file 1 — (PDF 94 kb) [file 122_2021_3779_MOESM1_ESM.pdf]

Theoretical and Applied Genetics

Early prediction of biomass in hybrid rye based on hyperspectral data surpasses genomic predictability in less-related breeding material

Rodrigo José Galán[1], Angela-Maria Bernal-Vasquez[2], Christian Jebsen[2], Hans-Peter Piepho[3], Patrick Thorwarth[1,2], Philipp Steffan[4], Andres Gordillo[4], Thomas Miedaner[1].

[1] State Plant Breeding Institute, University of Hohenheim, 70593 Stuttgart, Germany.

[2] KWS SAAT SE, Grimsehlstraße 31, 37574 Einbeck, Germany.

[3] Biostatistics Unit, Institute of Crop Science, University of Hohenheim, 70593 Stuttgart, Germany.

[4] KWS LOCHOW GMBH, Ferdinand-von-Lochow Straße 5, 29303 Bergen, Germany.

Corresponding author:

Thomas Miedaner
e-mail: thomas.miedaner@uni-hohenheim.de
Telephone No.: +49 711 459-22690

Available ORCID of the authors:

Angela-Maria Bernal-Vasquez: https://orcid.org/0000-0003-0415-831

Patrick Thorwarth: https://orcid.org/0000-0003-4456-2358

Thomas Miedaner: https://orcid.org/0000-0002-9541-3726

Online resource 1

Phenotypic data analysis

The combined analysis across environments was conducted following model (1) from Galán et al. (2020). For the combined analysis across locations, this mixed model is as follows:

$$\begin{aligned} \gamma \;=\; & G{:}\,L + Y \\ & + L \cdot G + Y \cdot G + Y \cdot L + L \cdot Y \cdot G \\ & + ENV \cdot T + ENV \cdot T \cdot R + ENV \cdot T \cdot R \cdot B + e \end{aligned} \qquad (1)$$

where γ is the observed genotype performance, G denotes the genotypes, L the locations, Y the years, T the trials within environments ENV (equivalent to year-location combinations), R the replicates within trials, B the blocks within replicates, and e the error associated with the observation γ. Error, trial, block, and replicate variances were assumed heterogeneous among environments. In model (1), the dot operator (·) specifies crossed effects (A·B) and fixed and random terms are separated by a colon (:), with fixed terms appearing first (Piepho et al. 2003). Variance components and pairwise variances of genotype mean (BLUEs) differences (needed for heritability estimation) were estimated by restricted maximum likelihood (REML) for all random effects in model (1). This also holds for estimation of the genotypic variance (σ_g^2), which required an additional analysis fitting the above model with random genotypic effects. Significance of variance component estimates was tested by model comparisons using likelihood ratio tests (Stram and Lee 1994).

Within environments, BLUEs of genotypes were analyzed following model (2) also from Galán et al. (2020). This mixed model is described as follows:

$$\gamma \;=\; G{:}\,T + T \cdot R + T \cdot R \cdot B + e \qquad (2)$$

This model (2) differs from the first model (1) only in dropping the year and location main effects and corresponding interactions with genotypes. Variance components for single environments were estimated as described previously for model (1).

References for online resource 1 only

Galán RJ, Bernal-Vasquez A-M, Jebsen C, Piepho H-P, Thorwarth P, Steffan P, Gordillo A, Miedaner T (2020) Integration of genotypic, hyperspectral, and phenotypic data to improve biomass yield prediction in hybrid rye. Theor Appl Genet. doi: 10.1007/s00122-020-03651-8

Piepho H-P, Büchse A, Emrich K (2003) A hitchhiker's guide to mixed models for randomized experiments. J Agron Crop Sci 189(5):310–322

Stram DO, Lee JW (1994) Variance components testing in the longitudinal mixed effects model. Biometrics:1171–1177
