# Supplementary material for: Early prediction of biomass in hybrid rye based on hyperspectral data surpasses genomic predictability in less-related breeding material
Source: Theor Appl Genet. 2021 Feb 17;134(5):1409–22. doi: 10.1007/s00122-021-03779-1 (PMC8081675; doi:10.1007/s00122-021-03779-1)
Supplement: Supplementary file 2 — (PDF 499 kb) [file 122_2021_3779_MOESM2_ESM.pdf]

Theoretical and Applied Genetics

Early prediction of biomass in hybrid rye based on hyperspectral data surpasses genomic predictability in less-related breeding material

Rodrigo José Galán[1], Angela-Maria Bernal-Vasquez[2], Christian Jebsen[2], Hans-Peter Piepho[3], Patrick Thorwarth[1,2], Philipp Steffan[4], Andres Gordillo[4], Thomas Miedaner[1].

[1] State Plant Breeding Institute, University of Hohenheim, 70593 Stuttgart, Germany.

[2] KWS SAAT SE, Grimsehlstraße 31, 37574 Einbeck, Germany.

[3] Biostatistics Unit, Institute of Crop Science, University of Hohenheim, 70593 Stuttgart, Germany.

[4] KWS LOCHOW GMBH, Ferdinand-von-Lochow Straße 5, 29303 Bergen, Germany.

Corresponding author:

Thomas Miedaner
e-mail: thomas.miedaner@uni-hohenheim.de
Telephone No.: +49 711 459-22690

Available ORCID of the authors:

Angela-Maria Bernal-Vasquez: https://orcid.org/0000-0003-0415-831

Patrick Thorwarth: https://orcid.org/0000-0003-4456-2358

Thomas Miedaner: https://orcid.org/0000-0002-9541-3726

Online resource 2

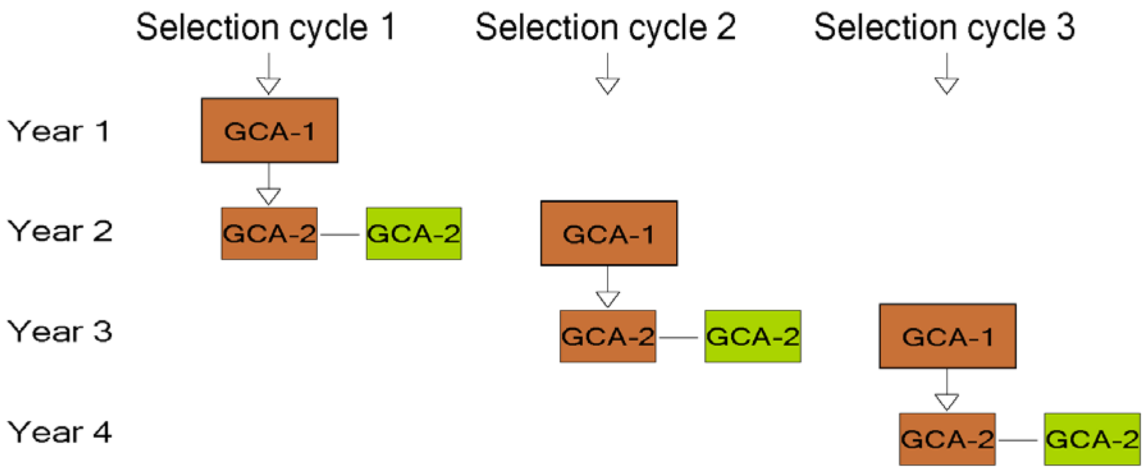

Supplementary Fig. S1 Schematic representation of the selection cycles in hybrid rye breeding program (adapted from Bernal-Vasquez et al. 2017). For across-cycles prediction, phenotypic, molecular, and hyperspectral data for model training could be collected in the second general combining ability trials (GCA-2), whereas prediction and validation are performed among the first GCA trial (GCA-1) and GCA-2 of a subsequent selection cycle, respectively. Brown boxes stand for grain yield trials, while green boxes for biomass trials.

Reference: Bernal-Vasquez A-M, Gordillo A, Schmidt M, Piepho H-P (2017) Genomic prediction in early selection stages using multi-year data in a hybrid rye breeding program. BMC Genet 18(1):51. doi: 10.1186/s12863-017-0512-8

Parental line B

Parental line A	Line 1	Line 2	Line 3	Line 4	Line 5	Line 6	Line 7	Line 8	Line 9	Line 10
Line 1		**30**								**26**
Line 2			**32**							
Line 3				**32**						
Line 4					**4**					
Line 5						**30**				
Line 6							**24**			
Line 7								**30**		
Line 8									**28**	
Line 9										**28**
Line 10										

Supplementary Fig. S2 Schematic representation of the single-round robin design used in the present study. The F1 plants (*n*=264) were derived from each of the chain crosses (shaded cells showing the size of each bi-parental family). Adapted from Verhoeven *et al.* (2006)

Reference: Verhoeven KJF, Jannink JL, McIntyre LM (2006) Using mating designs to uncover QTL and the genetic architecture of complex traits. Heredity 96(2):139–149

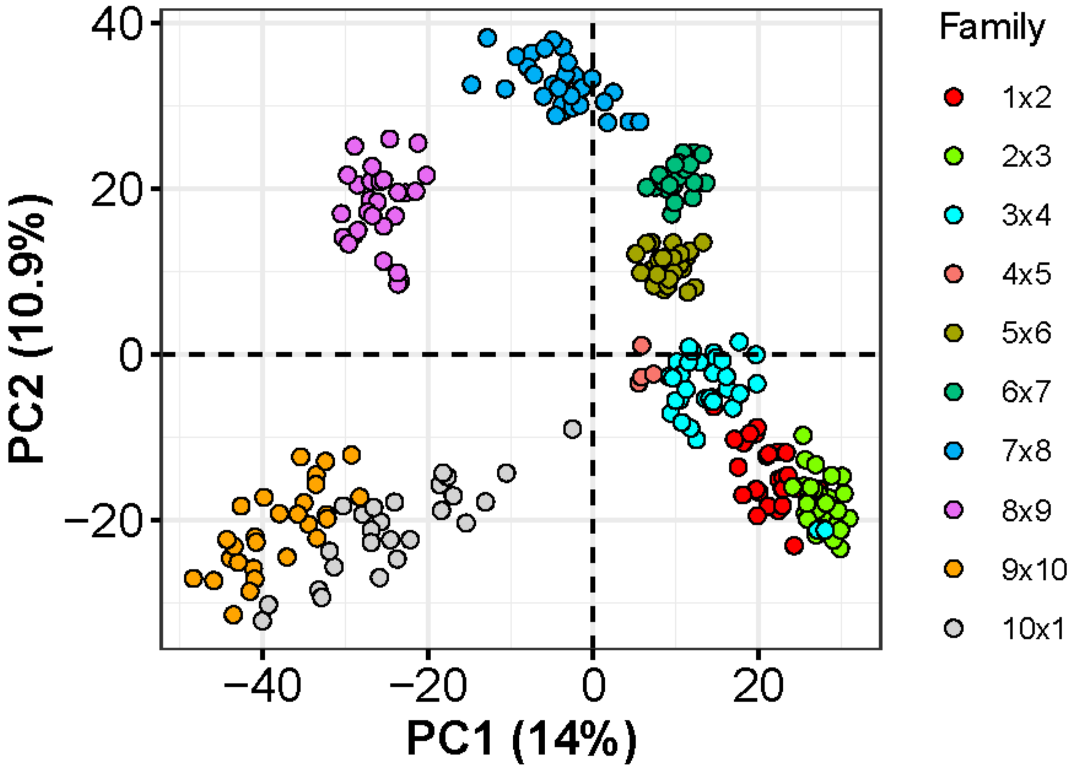

Supplementary Fig. S3 Principal component analysis (PCA) of the ten bi-parental families based on SNP data showing the outcome for the first two principal components.

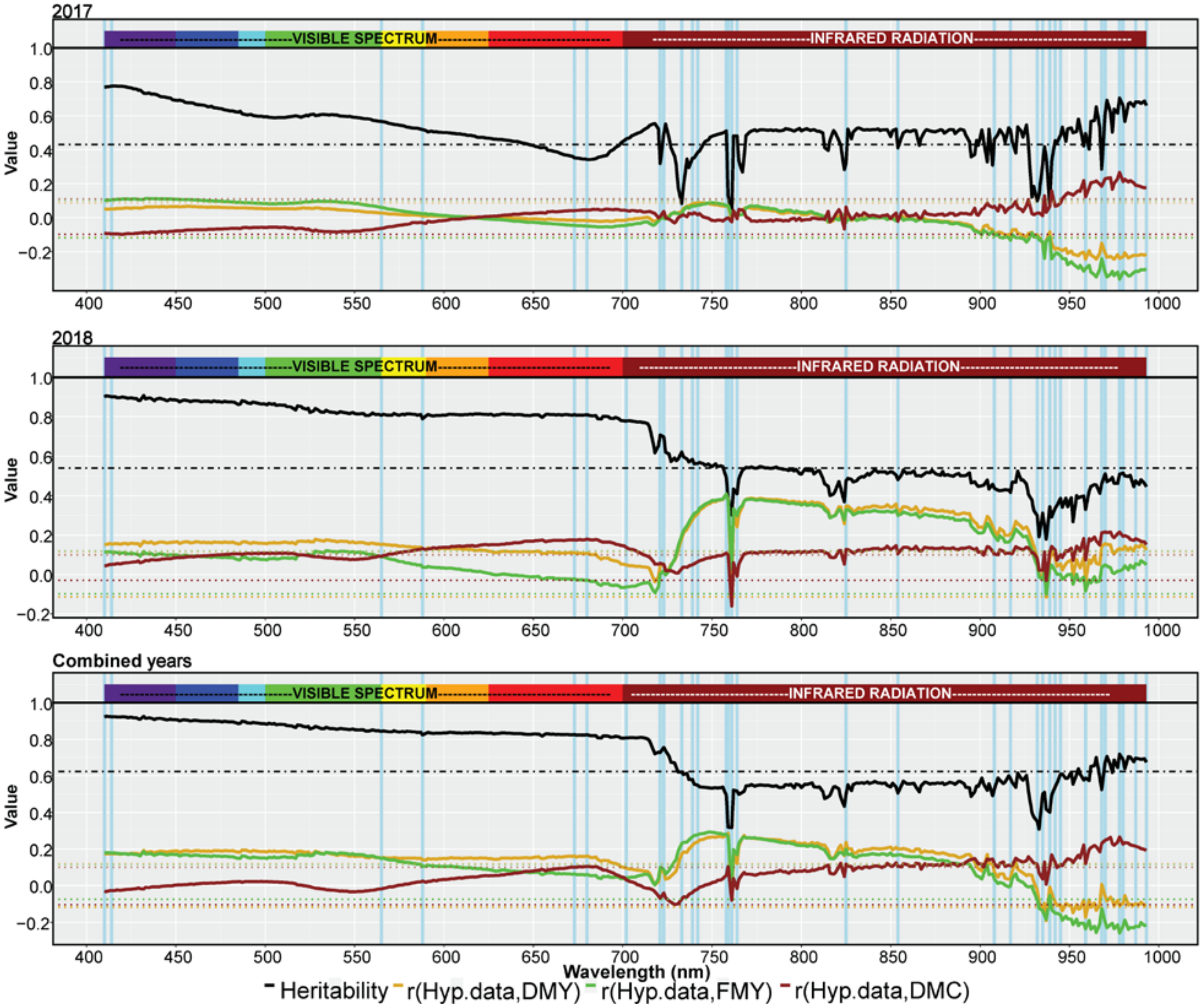

Supplementary Fig. S4 Heritability estimates (black line) for the hyperspectral bands, phenotypic correlations (r) between hyperspectral bands and dry matter yield (yellow line), fresh matter yield (green line), and dry matter content (brown line), and selected hyperspectral bands after the least absolute shrinkage and selection operator (Lasso, light blue vertical lines) for 274 winter rye hybrids assessed in two years, which were individually and combined analyzed. The mean heritability across selected wavelengths is denoted by the dot-dashed black line. Correlation estimates (in absolute values) ≥ to the respective dotted lines are significant ($p<0.05$).
